# Context-dependent regulation of feeding behaviour by the insulin receptor, DAF-2, in *Caenorhabditis elegans*

**DOI:** 10.1007/s10158-016-0187-2

**Published:** 2016-05-21

**Authors:** James Dillon, Lindy Holden-Dye, Vincent O’Connor, Neil A. Hopper

**Affiliations:** Centre for Biological Science, University of Southampton, Highfield Campus, University Road, Southampton, Hants SO17 1BJ UK

**Keywords:** *C. elegans*, Insulin, Behavioural plasticity, Electropharyngeogram

## Abstract

Insulin signalling plays a significant role in both developmental programmes and pathways modulating the neuronal signalling that controls adult behaviour. Here, we have investigated insulin signalling in food-associated behaviour in adult *C. elegans* by scoring locomotion and feeding on and off bacteria, the worm’s food. This analysis used mutants (*daf*-*2*, *daf*-*18*) of the insulin signalling pathway, and we provide evidence for an acute role for insulin signalling in the adult nervous system distinct from its impact on developmental programmes. Insulin receptor *daf*-*2* mutants move slower than wild type both on and off food and showed impaired locomotory responses to food deprivation. This latter behaviour is manifest as a failure to instigate dispersal following prolonged food deprivation and suggests a role for insulin signalling in this adaptive response. Insulin receptor *daf*-*2* mutants are also deficient in pharyngeal pumping on food and off food. Pharmacological analysis showed the pharynx of *daf*-*2* is selectively compromised in its response to 5-HT compared to the excitatory neuropeptide FLP-17. By comparing the adaptive pharyngeal behaviour in intact worms and isolated pharyngeal preparations, we determined that an insulin-dependent signal extrinsic to the pharyngeal system is involved in feeding adaptation. Hence, we suggest that reactive insulin signalling modulates both locomotory foraging and pharyngeal pumping as the animal adapts to the absence of food. We discuss this in the context of insulin signalling directing a shift in the sensitivity of neurotransmitter systems to regulate the worm’s response to changes in food availability in the environment.

## Introduction

An animal must be capable of adapting multiple behaviours in response to changes in its metabolic and nutritional status (Friedman 2010). These behavioural adaptations include changes in food intake and food seeking. Insulin signalling in the nervous system of vertebrates regulates feeding and food-associated behavioural plasticity (Kleinridders et al. 2014). In *Caenorhabditis elegans,* behavioural paradigms have been developed that model food-associated behavioural plasticity. *C. elegans* eat bacteria via the pharynx. Presentation of food triggers sensory inputs that drive an increased pumping that draws bacteria into the gut. This integrative response involves several transmitter pathways but is largely dependent of 5-HT, acetylcholine and neuropeptides (Franks et al. 2006; Dallière et al. 2016). In contrast upon the removal of food, worms initially display a reduced pumping rate; however, in the continued absence of food, the behaviour appears to undergo a contextual adaptation in which the pumping rate increases with time after food removal (Avery and Horvitz 1990; Dallière et al. 2016). Detailing the genetic determinants of these food-dependent behaviours highlights the central role of stimulation of pumping to food presentation but an unexpected permissive off food response that represents more than a simple loss of the activation that occurs on food. Indeed the genetic and time dependence of the off food response reveals an interplay between inhibition and excitation that actively controls the reduced pharyngeal pumping off food (Dallière et al. 2016). Further evidence for a shifting tone in pharyngeal pumping comes from observations that show a sensitization of the pharynx to food exposure subsequent to the protracted incubation to periods off food (Avery and Horvitz 1990; Lemieux et al. 2015). Taken together, these observations indicate complex regulation of food-dependent pharyngeal behaviours in which the intrinsic and extrinsic pharyngeal nervous system is involved.

*C. elegans* also displays adaptive locomotory behaviour to the changing availability of food. In the presence of food, *C. elegans* displays a ‘dwelling’ behaviour, which is characterized by slow forward and backward movement and a relatively high reversal frequency (Wakabayashi et al. 2004; Gray et al. 2005). Upon removal from food, worms adopt an area-restricted search strategy (ARS), which is characterized by an increased speed of movement and an increased number of turns (Gray et al. 2005). Extending the time that worms are left off food triggers a ‘dispersal’ behaviour where they move quickly and suppress turns to execute long straight forward runs (Hills et al. 2004; Wakabayashi et al. 2004).

In the case of both pharyngeal and locomotory function, the core transmitters that regulate behaviour are well understood. In addition, how modulation of these core behaviours impose adaptation to differing food cues is increasingly investigated and shown to engage neuropeptide transmitters including FLP, NLP and INS peptides (Pierce et al. 2001; Holden-Dye and Walker 2013; Cheong et al. 2015). The role of the insulin-like signalling pathway is consistent with the established way its signalling regulates the development in a food-dependent manner. This includes both *C. elegans* dauer formation, which is a developmentally arrested alternative third-stage larva that occurs during starvation and L1 arrest, where post embryonic development ceases if food is not available (Lau and Chalasani 2014). However, insulin-like signalling has been implicated in neural and behavioural plasticity particularly those that are modulated during some of the more acute changes in food availability. These insulin signalling-dependent changes in the adult are well described in classic learning paradigms in which food deprivation led to insulin-dependent plasticity (Sasakura and Mori 2013).

The components of the insulin signalling pathway are highly conserved (Murphy and Hu 2013). In *C. elegans,* they include DAF-2, a homologue of the insulin/IGF receptor and a number of down-stream signalling components that act as positive and negative regulators of insulin signalling, the latter including DAF-18 (Ogg and Ruvkun 1998). In *C. elegans,* there appears to be only one insulin-like growth factor receptor that acts widely as the downstream target for a large number of insulin-like ligands defined by the insulin-like growth factor family (Lau and Chalasani 2014). The utilization of distinct DAF-2 temperature-sensitive mutant lines has allowed investigators to probe for insulin dependence of behaviour with varying levels of signalling (Gems et al. 1998). This has led to the classification of DAF-2 alleles into two overlapping groups, denoted as Class I and Class II, which specify two distinct functions of the DAF-2 insulin receptor. Class I alleles tend to present less severe phenotypes that include constitutive formation of dauer larvae (*daf*-*c*), increased adult longevity (*age*), increased intrinsic thermotolerance (*itt*) and exhibit low levels of L1 larval arrest. In contrast, the phenotypes associated with Class II alleles are more severe and include all of the Class I phenotypes as well as some or all of the following: reduced adult motility, abnormal adult body and gonad morphology, high levels of embryonic and L1 larval arrest, production of progeny late in life and reduced brood size. Further detailed analysis of *daf*-*2* alleles has led to them being ranked according to the severity of these phenotypes (Patel et al. 2008; Nanji et al. 2005). Investigation of the predicted biochemical consequences of the mutants in the *daf*-*2* allelic series suggests that Class II mutants are more severely retarded in their signalling capacity than Class I. In particular, Class II mutants are disrupted in core insulin binding of intracellular tyrosine kinase signalling. In contrast, Class I mutants may retain efficacy of subclasses of insulin ligands or represent receptors with reduced signalling based on lower surface expression (Patel et al. 2008). This suggests potential complex background tone in insulin signalling, an observation reinforced by the differential ability of critical negative regulators to modulate the signalling (Patel et al. 2008).

Here, we use three *daf*-*2* alleles to conduct our behavioural analysis, which are representative alleles with differential impact on *daf*-*2* signalling. The Class I allele (*m577*) is predicted to encode a receptor with reduced surface expression that remains competent to signal some or a subset of insulin-like ligands. Further, we investigated two distinct Class II alleles (*e1370*) and (*e979*) which encode a receptor deficient in insulin binding and disrupted in their intracellular tyrosine kinase activity, respectively (Patel et al. 2008). Out study also utilized *daf*-*18*(*nr2037*) alleles, which lack the PTEN-associated phosphatase activity that dephosphorylates PIP3, which is the primary signal produced after DAF-2 activation to recruit downstream signalling. Acting to remove the DAF-2 signal makes *daf*-*18* an important negative regulator of *daf*-*2* signalling in *C. elegans* and is the best studied PTEN pathway in *C. elegans* (Liu and Chin-Sang 2015). These investigations show that the insulin-like signalling pathway regulates food-related behavioural plasticity in the adult *C. elegans.* We identify roles for the acute use of insulin signalling in response to food presentation and removal. The adaptive responses expressed at the level of locomotion and pharyngeal pumping are disrupted in *daf*-*2* deficient backgrounds indicating that insulin pathways are involved in the adaptive response to reduced food. These observations highlight an important contribution of insulin peptides to adaptive behaviours involving modulation of signalling in the mature nervous system.

## Materials and methods

### *C. elegans* culture and strains

Worm strains were grown and handled according to procedures previously described (Brenner 1974). The wild-type strain used was *C. elegans* variant Bristol, strain N2. Mutant strains used were DR1567 *daf*-*2*(*m577*)*III*, DR1563 *daf*-*2*(*e1370*)*III*, *daf*-*2*(*e979*)*III* and NS3227 *daf*-*18*(*nr2037*)*IV* and were provided by the CGC, which is funded by NIH Office of Research Infrastructure Programs (P40 OD010440). The peptide FLP17A (sequence KSAFVRFamide) was synthesized by Southampton Polypeptides Ltd (Southampton, UK), to [90 % purity]; 5-hydroxytryptamine creatinine sulphate was obtained from Sigma (Poole, UK).

### Behavioural assays

Young adult animals staged at L4 plus 1 day were first observed on plates seeded with OP50 bacteria and then transferred onto a fresh nematode growth medium (NGM) plate without food for 1 min, to remove bacteria from the worm cuticle. Worms were then transferred onto another fresh NGM plate without food and observations began 5 and 60 min after worms were transferred, with the plate lid closed. Animals in on food experiments were paired with the off food for 5- and 60-min experiments, and all experiments were performed at approximately 25 °C as previously described (Dillon et al. 2013).

### Measuring pumping rate in intact worms

Pharyngeal pumping was recorded by counting the contractions of the pharyngeal terminal bulb grinder using a hand counter. A full contraction and relaxation of the radially orientated muscle of the terminal bulb was defined as a single pharyngeal pump. The number of pharyngeal pumps was counted for a 15-s time period, and this was then used to calculate the pharyngeal pump rate.

### Measuring speed of locomotion

The number of body lengths travelled by an individual worm was counted for 15 s using a hand counter and this was then used to calculate the body lengths per minute. The average body length of an L4 plus 1-day old worm was measured as 1.4 mm based upon the measurement of ten individuals. The average body length was multiplied by the number of body lengths moved per minute to calculate the speed of locomotion expressed as µm s^−1^.

### Measuring the length of forward duration

The duration of forward movement was assayed by recording the amount of time the worm moved in a forward direction during a defined observation period. This was done eight times for each individual animal, and the average of these eight measurements was taken as the mean duration of forward movement for that individual. Statistical analysis was performed on the mean for each animal with the number of trials defined as the number of animals in the analysis.

### Electrophysiological assays

Young adult worms staged at L4 plus 1 day were either immediately transferred from an OP50 seeded NGM plate to a dish containing 3 ml of Dent’s saline or they were transferred to a non-seeded NGM plate for 60 min before being transferred to the dish containing Dent’s saline (composition in mM; d-glucose 10 mM, HEPES 10, NaCl 140, KCl 6, CaCl_2_ 3, MgCl_2_ 1, pH 7.4 with NaOH), supplemented with 0.01 % bovine serum albumin. A razor blade was used to make a transverse cut at the pharyngeal intestinal valve, typically within 2–5 min of being transferred to the Dent’s saline. The dissected pharyngeal preparation was transferred to a recording chamber containing Dent’s saline using a 10-μl pipette. Electropharyngeogram (EPGs) were recorded according to the method previously described (Dillon et al. 2013). The addition and duration of drug application is indicated in individual figures and legends. All drugs that were applied had been diluted to the required concentration in Dent’s saline. The solutions in the recording chamber were exchanged using a 1-ml pipette. The EPG recordings were made at 22–25^o^ C and when worms were not being used for recordings, they were returned to an incubator at 25.5 °C. The number of pumps performed during the application of each solution was counted and used to calculate the average rate of pharyngeal pumping. For each individual worm tested in the EPG, the pharyngeal pump rate (pumps/min) was calculated for the basal period of the EPG (10 min in Dent’s saline) and for the period of drug application (2.5 min 5-HT). The change in the number of pumps/min was then calculated by subtracting the basal rate of pumping from the pump rate during the drug application.

## Results

### Analysis of locomotory behaviour in *daf*-*2* mutants on and off food

We compared wild-type (N2) and *daf*-*2* mutants for locomotory behaviour on food. This was followed by removing worms from food and measuring behaviour 5 or 60 min after being placed on a no-food arena. As previously reported, strong *daf*-*2* mutants (*e1370* and *e979*) were lethargic on food and moved much slower than wild-type and *daf*-*2*(*m577*) worms (Table 1) (Gems et al. 1998; Gray et al. 2005). The weaker *daf*-*2*(*m577*) mutant had a speed that was indistinguishable from N2, whereas *daf*-*2*(*e1370*) moved at 51 % of N2 speed and *daf*-*2*(*e979*) did not move without first being touched (Table 1). After 5-min off food, wild-type animals increased their speed of movement and an increase was also observed for all *daf*-*2* mutants tested. After 60-min off food, the speed of wild-type worms is very similar to that measured after 5-min off food (210 ± 16.46 and 233 ± 15.4 μm/s, respectively). In contrast, the strong *daf*-2 mutants moved at a slower speed at 60-min off food compared to 5-min off food [~59 % decrease for *daf*-*2*(*e1370*) and 100 % for *daf*-*2*(*e979*)]. At the same time point, *e1370 ande979* moved 24 % and 60 % slower than the wild type and 15 and 55 % slower than the weak *daf*-*2*(*m577*) mutant, respectively.

**Table 1:** Rate of movement of wild-type N2 and *daf*-*2* mutant *C. elegans* on food and at increasing times after removal of food

Strain	Speed on food	Speed off food 5 min	Speed off food 60 min
N2	115 ± 14.66	233 ± 15.4	210 ± 16.46
*daf*-*2*(*m577*)	118 ± 17.75	208 ± 16.47	182 ± 26.21
*daf*-*2*(*e1370*)	58 ± 24.32*	177 ± 13.21*	72 ± 20.97***
*daf*-*2*(*e979*)	32 ± 19.63**	93 ± 16.44***	0

Animals with severe, Class II, *daf*-*2* mutations move significantly slower than wild-type animals on food. After 5-min wild-type animals increase their speed of movement and this is also seen in all *daf*-*2* mutants tested. After 60-min off food, *daf*-*2*(*e1370*) and *daf*-*2*(*e979*) both move much slower than wild-type and *daf*-*2*(*m577*) worms. Speed is calculated at µm/sec ± s.e.m assuming average worm length of 1.4 mm. Speed was compared between wild-type and *daf* mutants using an unpaired Student’s *t* test

* *p* < 0.05, ** *p* < 0.01, *** *p* < 0.001

We also determined the length of average forward duration of movement on food and off food 5 and 60 min after removal from food (Table 2). The mutant *daf*-*2*(*e979*) could not be accurately scored as it would only move upon prodding. The mean forward duration of wild-type animals increased after 60 min after being removed from food relative to the 5-min time point. This switch in foraging behaviour which reflects a change from local area search to dispersal (Gray et al. 2005) was lost in *daf*-*2*(*e1370*) mutants. This would suggest that this temporally regulated behavioural adaptation to food deprivation is dependent on an insulin signal which is deficient in *daf*-*2*(*e1370*).

**Table 2:** Wild-type N2 and *daf*-*2* mutant forward duration at increasing times after removal of food

Strain	Mean forward duration off food 5 min	Mean forward duration off food 60 min
N2	26.9 ± 2.4	46 ± 8.0*
*daf*-*2*(*m577*)	25.4 ± 3.4	23.9 ± 2.6
*daf*-*2*(*e1370*)	31.4 ± 5.3	33.1 ± 7.2

The mean duration (sec) of forward movement of wild-type worms (*n* = 30) increased after 60-min off food. This is not seen in *daf*-*2*(*m577*) (*n* = 15) or *daf*-*2*(*e1370*) (*n* = 13). Values are mean ± s.e.m. Mean forward duration was compared between off food for 5 min and 60 min using a paired Student’s *t* test

* *p* < 0.05

### Analysis of pharyngeal pumping behaviour in *daf*-*2* mutants on and off food

**Fig. 1:**
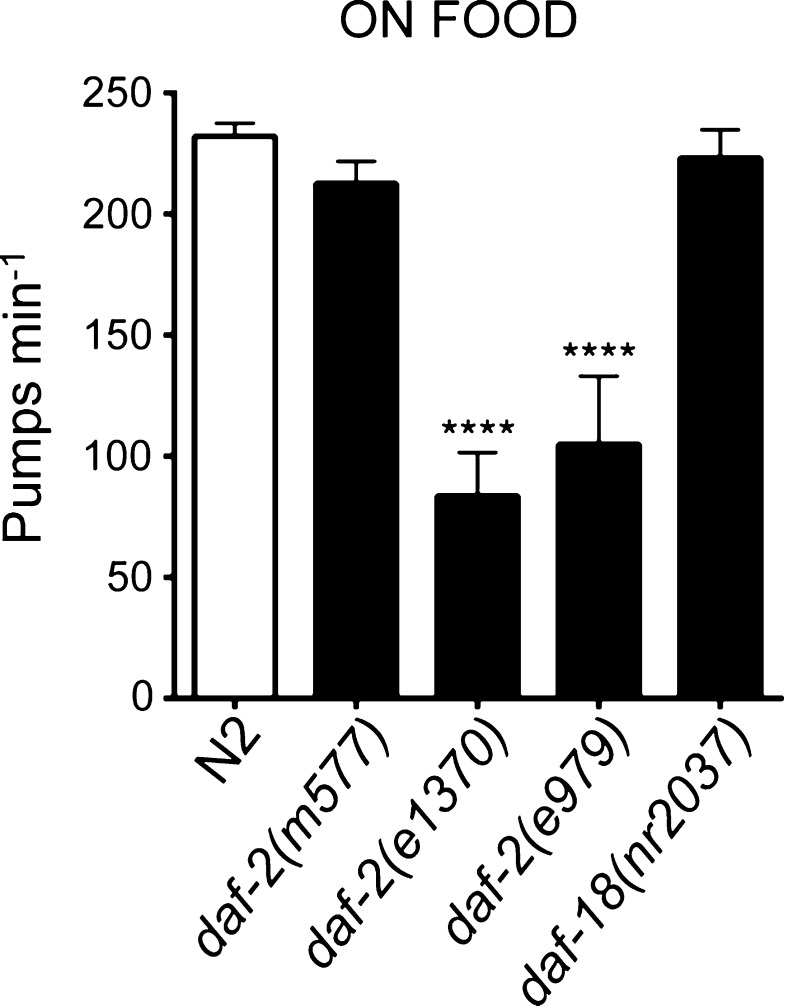
Pharyngeal pumping of *daf*-*2* and *daf*-*18* mutant worms on food. Class II, strong, *daf*-*2* mutations *daf*-*2*(*e1370*) (*n* = 12) and *daf*-*2*(*e979*) (*n* = 7) pump less well than N2 animals (*n* = 25) on food. The weak mutation *daf*-*2*(*m577*) (*n* = 17) and mutation in *daf*-*18*(*nr2037*) (*n* = 18) pump at >90 % of the rate shown by wild-type animals. *Error bars* represent standard error of the mean (s.e.m); asterisks mark comparisons different to wild-type N2 at ***p < 0.001 by one-way ANOVA with Bonferroni post-test

On food *e1370* and *e979daf*-*2* mutant alleles pumped at a rate 50 % less than that of wild-type animals on food (*p* < 0.001 compared to N2) (Fig. 1). *daf*-*2*(*m577*), on the other hand, exhibited a rate that was not significantly different from wild type. An independent route to manipulate the strength of insulin signalling is to investigate *daf*-*18,* mutants deficient in the PTEN activity that negatively regulates signalling downstream of *daf*-*2. daf*-*18* appears not to affect the response of the pharynx in the presence of food as worms with the *daf*-*18*(*nr2037*) null mutation pumped at a rate similar to the wild-type animal (Fig. 1).

It has previously been shown that insulin ligands of the DAF-2 signalling pathway are expressed in response to bacterial sensory cues (Li et al. 2003). This suggests the reduced pumping rate of Class II *daf*-*2* mutants may either be because of a lack of insulin signalling that promotes pharyngeal pumping in the presence of bacteria; or alternatively adult Class II *daf*-*2* mutants may not be able to pump at the same rate as wild type due to a non-specific physical impairment.

**Fig. 2:**
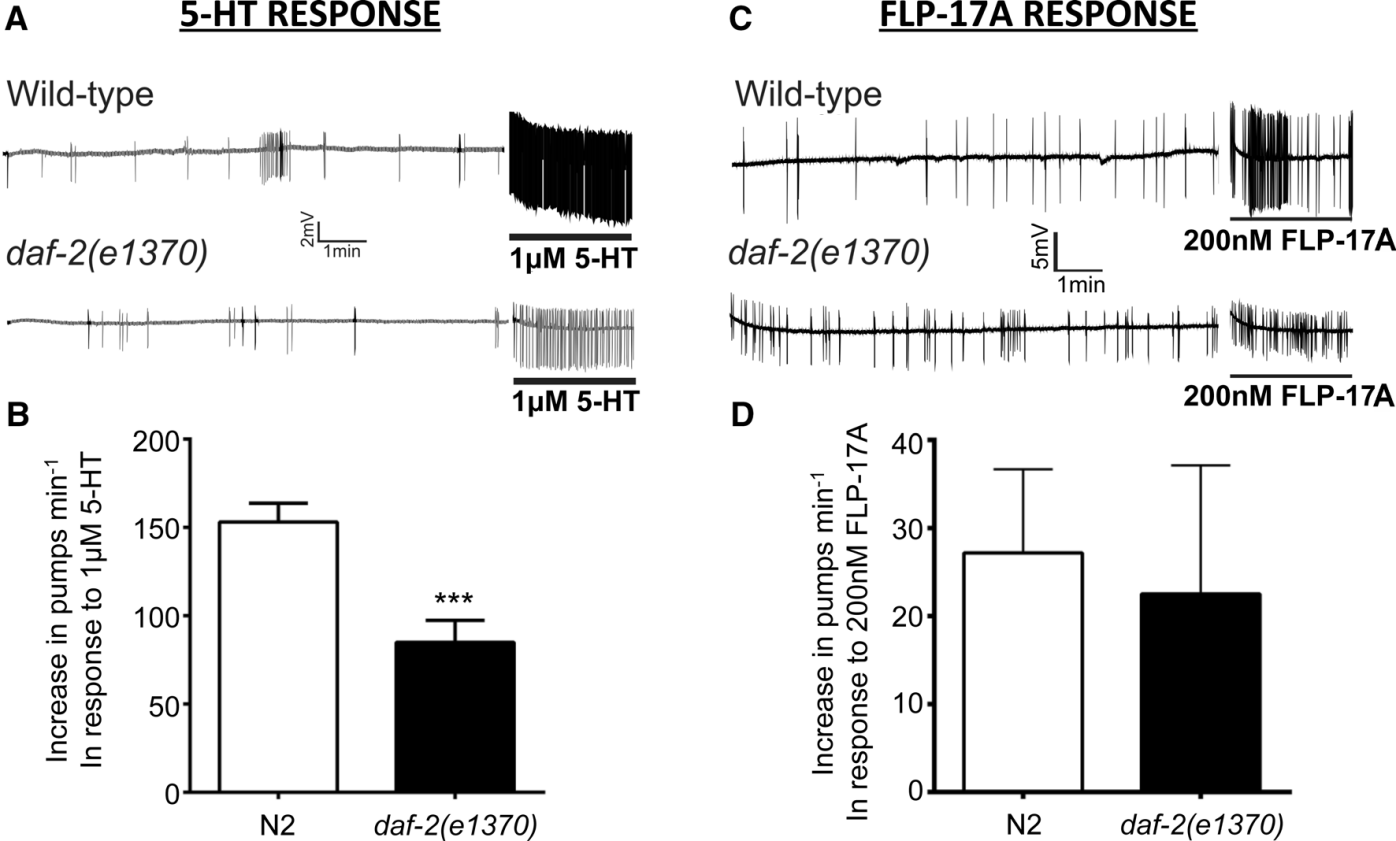
EPG recordings of cut head preparations reveal a selective reduction of 5-HT sensitivity in Class II *daf*-*2* mutants. **a** Representative EPG recordings from wild-type and *daf*-*2*(*e1370*) worms, showing the response to 1 µM 5-HT. For each recording, the basal rate was recorded for 10 min in Dent’s saline and then 1 µM 5-HT was added for 2.5 min (indicated by the* solid black line*). **b** The application of 1 µM 5-HT increased the basal pumping rate of both N2 (*n* = 15) and *daf*-*2*(*e1370*) (*n* = 14) animals. However, the average increase in the basal pumping rate of *daf*-*2*(*e1370*) animals was significantly lower than wild-type animals (***p < 0.001 in an unpaired student’s *t*-test). **c** Representative EPG recordings of the wild-type and *daf*-*2*(*e1370*) response to 200 nM FLP-17A. Recordings consisted of 10-min recording of basal activity in Dent’s saline, followed by a 2.5-min application of FLP-17A (indicated by the *black bar*). **d** The increase in pump rate of N2 and *daf*-*2*(*e1370*) in response to FLP-17A was not significantly different. *Error bars* represent the s.e.m for *n* = 4, for both strains

To examine the latter possibility, we determined whether there were any pharyngeal defects by making electropharyngeogram (EPG) recordings from *daf*-*2*(*e1370*) worms. The electropharyngeogram is an extracellular electrophysiological assay that provides a read-out of the activity of the neuromuscular network that underlies co-ordinated pharyngeal pumping. Electropharyngeogram recordings were made from a semi-intact preparation of the pharynx (see methods). 5-HT is a potent modulator of the pharyngeal network and exogenous application causes an increase in the pharyngeal pumping rate and a decrease in the duration of the pump (Rogers et al. 2001). *daf*-*2*(*e1370*) mutants were significantly less responsive to 5-HT (1 μM) compared to wild type (approximately 55 % of the wild-type response) (Fig. 2a, b). This is unlikely to be due to a general defect in the functional integrity of the pharyngeal neuromuscular system of *daf*-*2*(*e1370*) mutants because the response to the neuropeptide FLP-17A, which has been reported to potently stimulate pharyngeal pumping (Papaioannou et al. 2005), was not significantly different to wild type (Fig. 2c, d). Taken together, this suggests that insulin signalling at the point of food presentation contributes to stimulatory signals that cause the pharynx to elevate and sustain a high pump rate in the presence of food, and moreover, this insulin signalling pathway differentially affects neuromodulatory pathways.

**Fig. 3:**
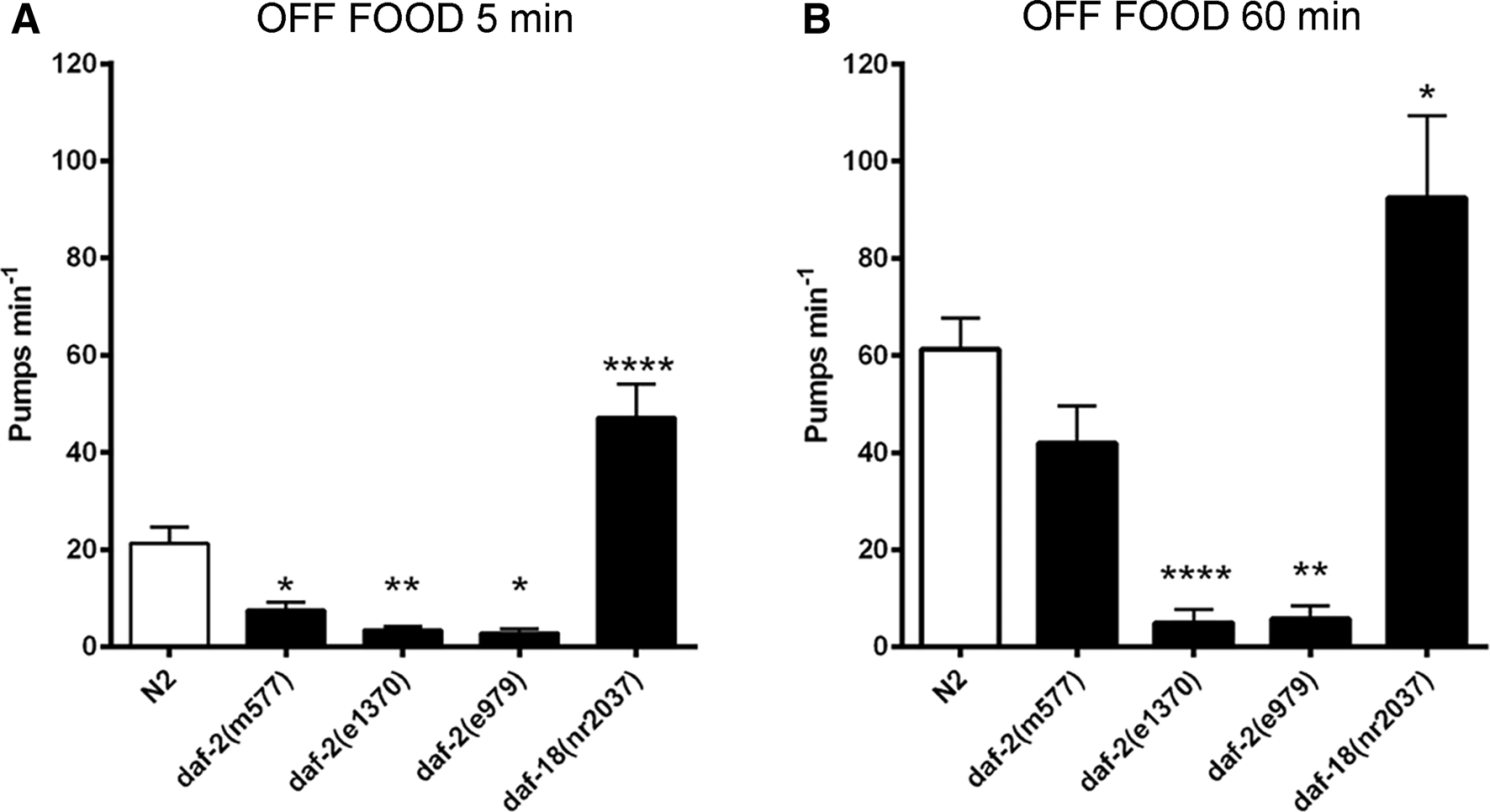
The role of insulin signalling in pharyngeal pumping off food. The indicated strains were removed from food, cleaned and observed 5 and 60 min after being placed onto a no food arena. **a** After 5-min off food, the *daf*-*2* mutants, *daf*-*2*(*m577*) (*n* = 17), *daf*-*2*(*e1370*) (*n* = 18) and *daf*-*2*(*e979*) (*n* = 7) displayed a reduced off food pump rate relative to N2 (*n* = 31). In contrast, the pumping rate of *daf*-*18*(*nr2037*) animals (*n* = 18) was significantly increased relative to N2. **b** When observed after 60-min off food, the pharyngeal pump rate of N2 (*n* = 31) increased relative to the 5-min time point. This time-dependent increase was observed in the Class I weak *daf*-*2* mutants, *daf*-*2*(*m577*) (*n* = 17). The more severe mutants, *daf*-*2*(*e1370*) (*n* = 18) and *daf*-*2*(*e979*) (*n* = 7) show no time-dependent increase in pumping relative to the same strains at 5 min. The elevated pharyngeal pumping rate of *daf*-*18*(*nr2037*) (*n* = 18) animals further increased after 60-min off food. *Error bars* represent s.e.m.; *asterisks* mark comparisons different to wild-type N2 at *p < 0.05, **p < 0.01 and ***p < 0.001 by one-way ANOVA with Bonferroni post-test

The role of *daf*-*2*-dependent insulin signalling in behavioural adaptation was further investigated in the context of pharyngeal pumping off food. Here, well-fed worms were removed from food, and their pump rate was measured 5 min after their removal and 60 min after they had been kept on agar plates lacking food (Fig. 3). The pharyngeal pumping rate of wild-type animals was reduced compared to on food but, as previously reported, this off food reduced pumping steadily increased over time off food (Dallière et al. 2016). In comparison, all the *daf*-*2* mutants showed a lower off food pump rate compared to wild type when measured 5 min after removal from food and in the strong alleles this reduction relative to N2 was sustained for the 60-min period of food deprivation. In contrast, after 60-min off food, the weak *daf*-*2* allele, *m577,* showed a similar progressive increase in pumping compared to wild type such that after 60 min it was not significantly different from N2. Taken together, this suggests that the regulatory tone that sets the off food pump rate is compromised in the *daf*-*2* background. Interestingly, the *daf*-*18* mutant that has enhanced insulin signalling shows an elevated off food pump rate 5 min after removal from food. This suggests it belongs to an increasingly well-recognized class of mutants that show constitutive pumping off food (Avery et al. 1993; Dallière et al. 2016). The reduced rate of pharyngeal pumping in the absence of food was more prominent in the Class II alleles suggesting that insulin signalling contributes positively to pharyngeal pumping even in the absence of bacteria and furthermore is required to drive the progressive increase in the off food pump rate.

**Fig. 4:**
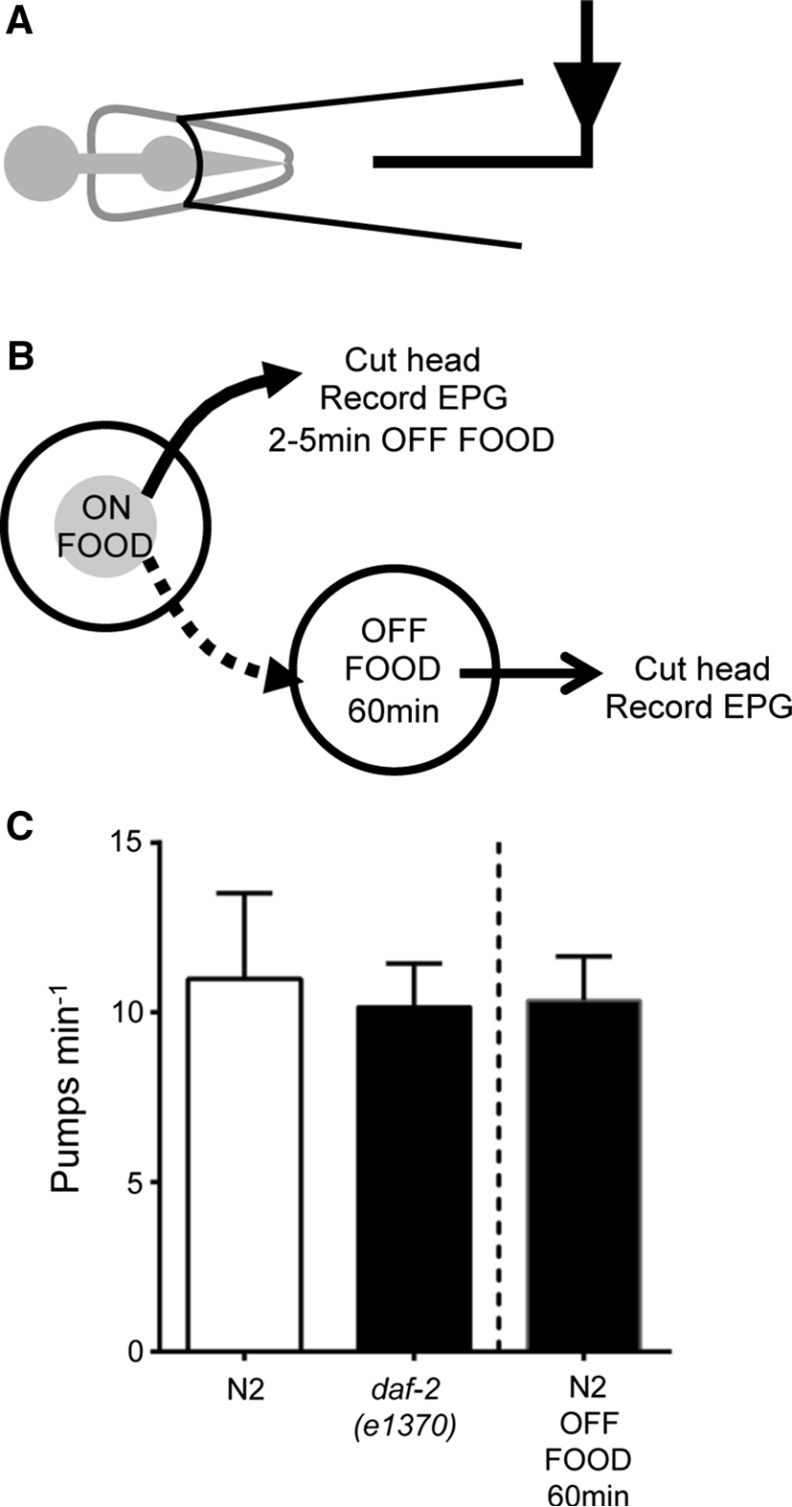
The mean pharyngeal pumping rate recorded from the cut-head prepared from intact worms 5 and 60 min after removal from food. **a** The EPG recording configuration from the cut-head pharyngeal preparation. **b** The experimental protocol. **c** The basal pumping rate of *daf*-*2*(*e1370*) (*n* = 34) and N2 (*n* = 37) animals was not significantly different after 5-min off food. After being removed from food for 60 min, the pharyngeal pumping rate of N2 animals (*n* = 32) did not increase. Pharyngeal pumping rate was compared between N2 and *daf*-*2*(*e1370*) using an unpaired Student’s *t-*test

There are a diverse range of insulin ligands that could mediate these responses and we were interested to investigate whether we could use the semi-intact configuration of the pharyngeal preparation to determine the source of the modulatory signal(s) that mediate this switch in pharyngeal behaviour, i.e. from an initial low rate of pumping to a higher rate, during food deprivation. To test this, we made EPG recordings from cut heads in which the preparation is relatively devoid of the systemic cues that will arise in the intact animal (Fig. 4a, b). These recordings were made from wild-type pharynxes 2–5 min after removal from food (Fig. 4c) and revealed that the pumping rate was similar to that of the intact animal in an off food context (Fig. 3a).

The fact that Class II *daf*-*2* alleles pump poorly off food and do not display the upregulation of pumping observed in the wild type after being off food for 60 min suggests that *daf*-*2* signalling acts to enhance late stage off food pumping. When worms that had been subjected to 60-min off food were subsequently tested in the EPG assay, they pumped at similar rate to intact worms after 5-min off food (Fig. 4c). This suggests that the progressive increase in pumping during prolonged food withdrawal involves a contribution from signals that are extrinsic to the pharynx. Interestingly, the EPGs that were recorded from the *e1370* cut-head preparation (Fig. 4c) identified that the cut-head pharynx actually pumps at a faster rate than the pharynx in the intact animal (10.2 vs 3.4 pumps min^−1^, respectively) after 5-min off food (Fig. 3a) again consistent with a role for an enhanced extrapharyngeal inhibitory signal in *daf*-*2*(*e1370*). The similar pump rate of dissected *e1370* and N2 pharynxes in the EPG (10.2 vs 11, respectively) further supports this possibility (Fig. 4c).

## Discussion

In the current study, we have investigated the contribution of insulin signalling to the adaptive behaviours associated with food availability. In keeping with previous experiments, we have used the switch from a replete food source to no food for 1 h to investigate ensuing adaptive responses (Dillon et al. 2013; Dallière et al. 2016). At the systems level of the whole animal, this paradigm is known to trigger modification of the worm’s sensory nervous system which is integrated into a network of downstream interneurons to modulate outputs and shift behaviour (Chalasani et al. 2007; Gray et al. 2005). Here, we investigated how this modified sensory drive, resulting from acute food deprivation, translates to changes in locomotion and pharyngeal pumping comparing wild-type responses with those of mutants with altered insulin signalling.

The behavioural pattern of *C. elegans* in terms of locomotion and foraging behaviour on and off food has been well characterized. On food worms move at a slower speed compared to off food and there is a well-established ‘switch’ in behaviour that occurs 30–40 min after removal from food (Hills et al. 2004; Gray et al. 2005). This latter behaviour reflects a progressive change in behaviour which is conveniently defined as two states that can be discriminated by a transition from a low duration of forward movement to a higher duration of forward movement. We scored the behaviour of insulin signalling mutants in these paradigms, and our observation that the locomotion of the severe alleles on food is markedly reduced is consistent with previous observations (Gems et al. 1998) that may reflect a developmental deficiency that impacts on core regulation of locomotion. In addition, we find that *daf*-*2* mutants e1370 and e979 move faster in the absence than in the presence of food i.e. similar to wild type. Indeed the fold increase in speed when food is removed is greater than the fold increase observed for wild type for this same transition. Nonetheless these mutants still fail to increase their speed of locomotion to a similar absolute value compared to wild type. Again, this may reflect a core deficit imparted during development of locomotory circuits in the insulin signalling mutants. However, closer inspection of the pattern of behaviour of the insulin signalling mutants during the period of food deprivation suggests a more central role for the insulin pathway in regulating behavioural transitions during food deprivation. This is indicated by the observation that whilst the *daf*-*2* deficient worms all increase their speed relative to their on food control when first moved off food they then fail to sustain this enhanced speed. Moreover, these mutants do not exhibit the transition to a high duration of forward movement after 60-min off food, a defining feature of dispersal behaviour in wild type. This indicates a contribution of *daf*-*2*-mediated insulin signalling in the transition from local area search to dispersal. Our data suggest that insulin signalling is required to switch between these behaviours. This context-dependent regulation and discrete dependence on insulin is consistent with observations made on the cellular responses of mutants deficient in selective members of the INS neuropeptide family (Leinwand and Chalasani 2013; Murphy and Hu 2013).

When we investigated the pharyngeal response to food and food deprivation across time, we identified a distinct contribution of insulin signalling. On food worms show a high pump rate that is much reduced upon removal from food but then undergoes an adaptive response reflected in a progressive increase in pumping over time (Dallière et al. 2016). Investigating transmitter mutants have identified important determinants of this behaviour. The high pumps rates on food are largely dependent on 5-HT stimulation of pumping that is driven by acetylcholine and glutamate modulation of pharyngeal muscle contraction-relation cycles. However, a number of other transmitters contribute to this and neuropeptides are important in maintaining the high pump rates that the worm sustains on food (Cheong et al. 2015). In the latter case, this dependence is readily observed by a clear deficiency in on food pumping seen in *egl*-*3* mutants (Dallière et al. 2016). These mutants are devoid of several classes of neuropeptides because *egl*-*3* encodes pre-proconvertase activity that processes NLP, FLP and INS peptides. Although in vitro investigations have revealed a number of candidate peptides involved in regulation of pharyngeal activity, there has been more limited investigation in vivo (Papaioannou et al. 2005; Rogers et al. 2001). These studies reveal that peptidergic regulation may impact on pharyngeal muscle excitability. The observations showing that *daf*-*2* deficient worms, particularly the severe alleles, are similarly deficient in the ability to maintain high rates of pumping on food suggests members of the INS neuropeptide family, acting through *daf*-*2,* contribute to the on food response (Dwyer and Aamodt 2013). The wide expression of *ins* peptides would allow these to function locally within the pharyngeal system or via systemic release from non-pharyngeal tissue including the nerve ring and intestine. Notably, although the sensitivity of the pharynx to the key modulator 5-HT is reduced, the intrinsic ability to exogenously drive pharyngeal pumping is not impacted as the peptide FLP-17 is as effective in increasing pumping in the N2 and *daf*-*2*(*e1370*) strain. Thus, our analysis of on food pumping is consistent with previous observations that insulin signalling is an important determinant of on food pharyngeal pumping and suggests INS-mediated signalling upregulates the sensitivity to 5-HT (Dwyer and Aamodt 2013).

In addition to the deficit in on food pumping in insulin signalling mutants, there is a change in their off food response relative to N2. Thus, the *daf*-*2* mutants are all significantly reduced in their off food pump rate, and the *daf*-*18* mutants are significantly enhanced in their off food pumping. These observations reinforce the conclusion made elsewhere that off food and during the subsequent food withdrawal, the reduced pharyngeal pumping rate is not simply loss of a positive cue. Rather, the shift of the worm to a no food context represents a discrete sensory cue with definable downstream circuits that finely tune pumping rate to the environmental context.

The low level of the *daf*-*2* pumping off food is similar to the previously reported off food pump rates seen in the *egl*-*3* mutants (Dallière et al. 2016). These rates are much lower than the N2 off food pumping and suggest a role for *egl*-*3* processed INS peptides in upregulating pharyngeal pumping during food deprivation.

In contrast to *daf*-*2* mutants, mutants for *daf*-*18*, the PTEN negative regulator of the DAF-2 pathway, exhibit an elevated and increasing pump rate off food relative to wild type which is similar to the constitutive pumping off food previously observed with *eat*-*4* and *unc*-*31* mutants (Dallière et al. 2016). The constitutive pumping of these mutants indicates that in the absence of food, there are signals that actively suppress pump rates. Because previous reports indicate that *eat*-*4* and *unc*-*31* constitutive pumping is additive (Dallière et al. 2016), there are at least two routes through which this active suppression of pumping off food can work. Taken together, these data suggest that a *daf*-*18*-dependent signal suppresses pumping off food and, given this is a negative regulator of DAF-2, is consistent with the *daf*-*2* data indicating a stimulatory role on pharyngeal pumping for insulin signalling off food. Thus, our analysis of *daf*-*2* and *daf*-*18* mutants off food suggests that insulin signal(s) may promote either the stimulatory pathway that drives off food pumping and is largely neuropeptide dependent or may contribute directly or via modulation to the pathways that suppress the off food pump rates through an interaction in a glutamate (*eat*-*4*) or an UNC-31 pathway.

The modulation of the pharyngeal pump rate in the off food context highlights that DAF-2 contributes to this behaviour (Dwyer and Aamodt 2013). The current observation highlights the previously unappreciated fact that DAF-18 contributes to this control. That these mutants act against the *daf*-*2* mutants loss of off food pump rates would be most parsimoniously explained by its known function as a negative regulator of DAF-2 signalling (Patel et al. 2008). This requires a careful double mutant analysis involving a series of *daf*-*2* alleles, as the penetrance of DAF-18 negative regulation is very dependent on the level of background insulin signalling (Patel et al. 2008). Interestingly, the *daf*-18 null mutant expresses a strong constitutive pump rate off food and based on current understanding implies a role in one or two pathways that are genetically resolved by their *eat*-*4* and *unc*-31 dependence. In view of the penetrant nature of *daf*-*18* mutant on the off food pumping investigating whether *daf*-*18* genetically interacts with these pathways will be informative. This will help define if insulin signalling acts on one or both off food pathways or if its activity is beyond the negative regulation of *daf*-*2* and involves PTEN-like modulation of glutamate or other neurosecretory signalling that remains a possibility based on observations from other organisms (Jurado et al. 2010). Further analysis of the interaction between these pathways will provide insight into which mechanism, or mechanisms, prevail.

**Fig. 5:**
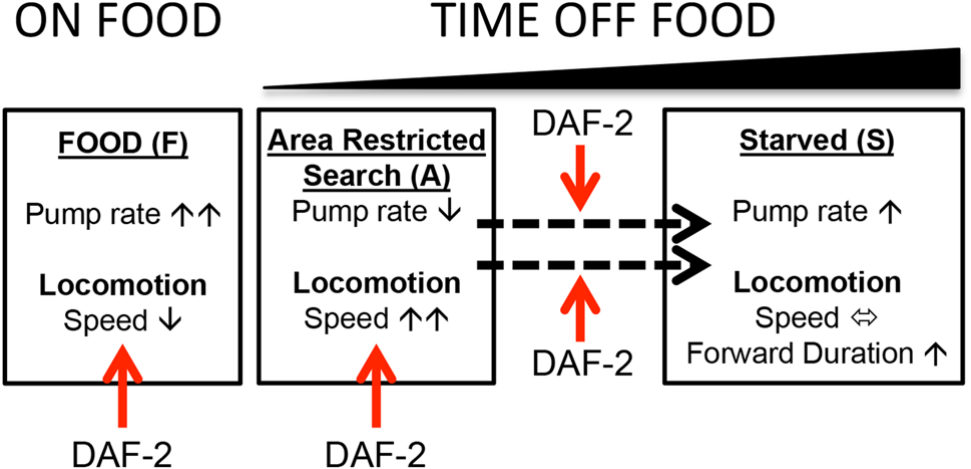
Schematic representation of insulin regulation of food-dependent behaviour. The adaptive changes in locomotion and feeding behaviour that occur under each condition are indicated by the* black arrows*. Insulin signalling, indicated by *red arrows*, modulates both behaviours on food and in the area-restricted search and it is required for the adaptation in both behaviours measured after 60-min off food. It is clear that both on and off food responses represent distinct contexts and the intersection of DAF-2-dependent signalling with the determinants of changing locomotion or pharyngeal function might be at the level of stimulation or inhibition of the circuits that organize these behaviours. An example would be the distinct role that *eat*-*4* and *unc*-*31* pathways play in pumping off food providing DAF-2 with distinct routes to selectively modulate off food pumping (Dallière et al. 2016) (color figure online)

Overall, the data reinforce previous observations that insulin signalling has a complex function in the adult nervous system (Zhao et al. 2004; Van Der Heide et al. 2005) and highlights how the pharyngeal system can be used to refine this understanding. As noted, investigating phenotype in mutants which may have important developmental effects can confound defining their role in the mature nervous system. However, as indicated by our observations, temporal changes in their behaviour highlight that insulin-dependent signalling contributes to adaptive behaviours in a context not easily explained by a global developmental impairment (Fig. 5). Further cellular investigation of the neural circuits underpinning this adaptive behaviour will serve to reinforce and elucidate the role played by insulin signalling in the response to food deprivation in adult *C. elegans*.

In the broader context, observations made in the mammalian system have highlighted the role of insulin in cellular mechanisms of neural plasticity that underlie learning and memory. For example, insulin has been shown to modulate the activity of excitatory ionotropic glutamate receptors and ionic receptors (Ahmadian et al. 2004). This would allow the insulin pathways to reconfigure nerve function. In *C. elegans,* there have been fewer measurements of neuronal excitability but the ability to modify excitability has been inferred by imaging studies and altered signalling detected in mutants deficient in specific peptides (Ohta et al. 2014; Oda et al. 2011; Kodama et al. 2006). In the current study, we show that network excitation is directly impacted by a global deficiency in insulin signalling to lend further weight to the notion that insulin in the adult nervous system provides a powerful route to control brain function.
